# Men matter: a cross-sectional exploration of the forgotten fathers of children born to adolescent mothers in South Africa

**DOI:** 10.1136/bmjopen-2024-092723

**Published:** 2025-07-03

**Authors:** Kathryn Steventon Roberts, Colette Smith, Lucie Dale Cluver, Elona Toska, Jane Kelly, Angelique Thomas, Janke Tolmay, Marguerite Marlow, Lorraine Sherr

**Affiliations:** 1 Department of Social Policy and Intervention, University of Oxford, Oxford, England, UK; 2 Institute for Global Health, University College London, London, England, UK; 3 Department of Psychiatry and Mental Health, University of Cape Town, Rondebosch, Western Cape, South Africa; 4 Centre for Social Science Research, University of Cape Town Faculty of Humanities, Rondebosch, South Africa; 5 Institute for Life Course Health Research, Department of Global Health, Faculty of Medicine and Health Sciences, University of Stellenbosch, Stellenbosch, Western Cape, South Africa

**Keywords:** Pregnancy, Family, HIV & AIDS, Community child health, MENTAL HEALTH

## Abstract

**Abstract:**

**Background:**

Fathers are intricately bound to the experience of adolescent mothers and their children. Yet, fathers of children born to adolescent mothers, particularly within the context of HIV, remain neglected in the literature. These exploratory analyses provide insight into the characteristics of fathers of children born to adolescent mothers affected by HIV in South Africa.

**Setting:**

Eastern Cape Province, South Africa.

**Design:**

Cross-sectional data from a prospective cohort study.

**Participants:**

Young mothers (10–24 years of age) and their children (0–68 months). All mothers completed detailed study questionnaires, including standardised and study-specific measures, relating to their self, their children and the fathers of their children. Summary statistics are presented based on maternal self-report of father characteristics. χ^2^ tests and t-tests (Fisher’s exact/Kruskal-Wallis tests, where appropriate) were additionally used to explore sample characteristics (including father characteristics, maternal experience and child characteristics) according to paternal age and father involvement in childcare (defined by responses to four maternal self-report questions). Father characteristics were also explored according to maternal HIV status and maternal mental health status.

**Results:**

40% of fathers were adolescents (10–19 years) at the birth of their children. Overall, father involvement was low (19.5%). Compared with noninvolved fathers, involved fathers were more likely to be older when their child was born (21 years vs 20 years, t=4.30, p=0.04), to be in a relationship with the mothers of their children (74.8% vs 47.2%, χ^2^=40.8, p≤0.0001), to reside with their children and their mothers (14.7% vs 3.7%, χ^2^=49.3, p≤0.0001) and to attend the first antenatal appointment (4.3% vs 1.5%, χ^2^=5.21, p=0.02). A quarter (25.4%; 227/894) of the adolescent mothers in the sample were living with HIV. The prevalence of maternal HIV was found to be higher among adolescent mothers of children born to older fathers compared with adolescent fathers (31.7% vs 15.9%, χ^2^=28.3, p≤0.001). Likewise, depressive symptoms were more prevalent among adolescent mothers of children born to older fathers compared with adolescent fathers (9.9% vs 5.3%, χ^2^=6.08, p=0.01). Adolescent mothers reporting poor mental health were less likely to be in a relationship with the fathers of their children (41.8% vs 54.1%, χ^2^=7.32, p=0.03) and more likely to experience domestic violence perpetrated by the fathers of their children (8.2% vs 3.3%, χ^2^=6.07, p=0.01) and to engage in arguments about finances with the fathers of their children (30.0% vs 17.0%, χ^2^=10.8, p=0.001). While some differences in individual subscales were identified, overall composite scores of child cognitive development did not differ according to father age or father involvement.

**Conclusions:**

Analyses provide the first preliminary description of the fathers of children born to adolescent mothers affected by HIV in South Africa. Fathers are inherently tied to the experiences of adolescent mothers and their children. Father involvement with their children was low. Further research is required to explore the potential barriers to father involvement and pathways to overcome these. Efforts to bolster father engagement, such as the inclusion of fathers within maternal and child service provision, may have benefits for fathers, adolescent mothers and their children. There was a high prevalence of adolescent fatherhood in the study. Adolescent fathers may have specific needs requiring tailored intervention for adolescent parent families. The need for the inclusion of fathers within policy, programming and research remains.

**STRENGTHS AND LIMITATIONS OF THIS STUDY**

- Data are drawn from a robust sample of adolescent mothers and offer the first detailed exploration of the characteristics of fathers of children born to adolescent mothers in the context of HIV in South Africa.
- A core strength of this study is the broad array of domains covered by the questionnaire and the use of validated and standardised scales.
- Data are drawn from adolescent mother reports and as such findings are confined to maternal views.
- Data are drawn from the Eastern Cape Province of South Africa, potentially restricting the generalisability of results beyond the region.

## Introduction

 Fathers are key to the experience of adolescent (10–19 years)^1^ motherhood. Recent decades have brought about change in the role and understanding of fathers.^2 3^ Yet, within the context of adolescent pregnancy, motherhood and HIV, fatherhood remains a neglected topic. Given the input of fathers within the experience of conception, pregnancy, parenting and across the life course of their children, such an absence of literature is a disservice, with possible far-reaching consequences for policy and programming.

Expected to reach 435 million by 2050, the adolescent population in sub-Saharan Africa is the fastest growing population in the world.^4^ As such, the needs of adolescents are focal to the success and potential of individuals and the region as a whole.^5^ Adolescents within the sub-Saharan African region face specific challenges including living within HIV-endemic communities^6^ and the highest rates of adolescent pregnancy globally.^7^ While there is an emerging body of evidence relating to adolescent mothers and their children,^8^,^12^ the complex needs of this population remain largely unexplored and unmet, and there is an absence of knowledge relating to fathers within this context.

It is well established that active father involvement has broad positive impacts on child^13^,^19^ (i.e., development, feeding), maternal outcomes (i.e., mental health) and prevention of mother-to-child transmission outcomes (i.e., antiretroviral adherence, viral suppression and HIV-free infant survival).^20^,^25^ The impacts of father involvement may differ based on certain characteristics of the father.^19^ Much of the existing literature on fatherhood focuses on adult fathers, with particular attention to age-disparate relationships when it comes to teenage mothers. This age disparity raises issues of concerns of abuse, transactional sex, increased HIV acquisition and child marriage.^26^,^29^ Hence, policy, programming and research efforts often bring attention to adolescent pregnancy within the context of age-disparate relationships. Given this focus, adolescent fathers are often excluded from research and policy agendas. Evidence in existence is often outdated, with much of the literature focusing on adolescent fathers published before 2000.^30^

In South Africa, one in three females will experience pregnancy during adolescence.^7^ Country estimates of adolescent fatherhood are difficult to ascertain, possibly due to unknown or a reluctance to share paternity status^31^ or the lack of routine documentation of father variables and noninclusion of fathers in most antenatal care. However, in some communities within South Africa, fathering a child signifies masculinity and adolescents may seek out fatherhood to affirm a masculine status.^32^ Both wanting to demonstrate masculinity and engaging in sexual risk-taking behaviours have been identified as factors contributing to adolescent fatherhood in South Africa.^32^ Challenges for adolescent fatherhood include financial dependency and caring responsibilities for children.^32^ Previous research in South Africa has highlighted the prevalent perception among fathers, and often mothers, that a father’s primary role is to provide maternal provision. If fathers cannot provide financially (especially due to high unemployment rates), this may lead to a sense of emasculation and inadequacy, prompting fathers to limit their involvement (of having their involvement limited) with their children.^33^ Possible solutions to navigating such challenges vary in the literature—adolescent fathers may rely on financial support for themselves and their children from their caregivers,^34^ or adolescent fathers may actively renegotiate their identity to take responsibility for their child(ren), restructuring goals and relationships to provide emotional and financial support for their child(ren).^35 36^ Father involvement (referring to the fathers of children born to adolescent mothers) may be a critical form of support for adolescent mothers and their children. Father presence may also interrupt intergenerational cycles of adolescent pregnancy and parenting, as father absenteeism within South Africa has previously been linked to early sexual debut and adolescent pregnancy.^37^

The high prevalence of adolescent pregnancy within South Africa is set against a backdrop of one of the highest HIV prevalence rates in the world.^6^ There is limited evidence relating to fatherhood within the context of families and children affected by HIV^38 39^ and an absence of evidence relating to adolescent fatherhood within the context of HIV. Among adults, compared with mothers not living with HIV, families of mothers living with HIV were more likely to experience paternal absence and disengagement.^40^ Previous investigations of fatherhood in the context of HIV have focused on fathers residing with their children and financial contributions with an absence of investigation into the activities undertaken by fathers within the lives of mothers and their children affected by HIV^38^ and the impact of fathers on maternal and child outcomes. Indeed, for the most part, the impact of fathers is most commonly documented when tracking paternal orphanhood with a focus on deceased fathers rather than on the living.^41^

### Objectives

This study uses data from adolescent mother reports to explore the characteristics of the fathers of children born to adolescent mothers and their role in the lives of adolescent mothers and their children. Analyses examine whether maternal and child characteristics differ according to paternal age or paternal involvement, and whether father characteristics differ according to key maternal characteristics including maternal HIV and maternal mental health.

## Methods

### Study design, setting, eligibility criteria

This study included data for 894 fathers based on adolescent mother reports. Data were drawn from a cross-sectional study of a cohort of young mothers (10–24 years; n=1046) residing in the Eastern Cape Province, South Africa. Participants were excluded from analyses if young mothers were not classified as adolescent mothers (who had given birth at 20 years of age and above; n=44), children were above 68 months of age (in keeping with the validated age range of the Mullen Scales of Early Learning^42^; n=23), the fathers were unknown to the mothers (hence no fatherhood reported data) or the father age was unknown (n=80). Mothers were asked to provide details about the biological fathers of their children. This did not include male partners who were not the biological fathers of their children. Second- and third-born children were excluded from analyses relating to child cognitive development (n=5). In total, n=894 adolescent mother-child dyads were included in the analyses. The sample size was based on year-on-year fertility rates for South Africa during the three waves of the existing Mzantsi Wakho study.^43^ A sample size of 220 was calculated based on this methodology; however, to ensure sufficient power for quasi-experimental longitudinal data analyses, the target sample size was n=1000.

Mothers with one or more living child(ren) were interviewed between March 2018 and July 2019. Mothers were recruited via six parallel sampling strategies developed with an advisory group of adolescent mothers and regional experts: health facilities, secondary schools, service provider referrals, maternity obstetric units, neighbouring adolescents of mothers and referrals from adolescent mothers enrolled in the study. Participants were included in the study if mothers were born between 1995 and 2007 and if they had been pregnant or had at least one child before the age of 19 years. All mothers (and caregivers; if participants were <18 years of age) provided informed written consent. Mothers completed detailed questionnaires (administered via electronic tablets by trained data collectors) consisting of validated scales and study-specific measures relating to health, relationships, community, HIV (if applicable), child development and parenting experience, and the fathers of their children. Participants completed all components of data collection in their language of choice (isiXhosa or English), and data were translated and back-translated as appropriate.

### Patient and public involvement

All data collection tools were piloted with adolescent mothers (n=9) and adolescents living with HIV (n=25).

###  Ethical approvals

Ethical approvals were obtained from the University of Cape Town (HREC 226/2017), University of Oxford (R48876/RE002) and University College London (14795/001). Additional local approvals and permissions were obtained from the Provincial Departments (Eastern Cape, South Africa) of Health, Education, and Social Development.

### Measures

Data were obtained from a range of self-report and standardised assessments.

### Fathers of children born to adolescent mothers

All data relating to the fathers of children born to adolescent mothers were obtained from maternal reports inclusive of paternal age (years), paternal HIV status, residential status, whether the father was the main caregiver of the child, domestic violence perpetration towards the mothers (adolescent mothers were asked if they had experienced domestic violence perpetrated by the fathers of their children during their pregnancy), paternal reaction to pregnancy and attendance at first antenatal appointment.

A measure of paternal engagement was generated. This was calculated from responses to four questions posed to adolescent mothers relating to *father involvement* in childcare: whether the fathers *help look after the child*, *buy things for the child*, *help with washing and food preparation,* and *spend time with the child* at least once every 2 weeks. These four questions were combined into a composite measure indicating father involvement (scored 0–4) with higher scores representative of involvement in more activities. The overall involvement score for the sample was 0.43 (SD: 1.03; scored 0–4), suggesting limited overlap between involvement categories. This composite measure was also split into a binary measure of any involvement (scoring on at least one of the above four measures) and no involvement (not scoring on any of the above four measures).

### Adolescent mothers

Maternal sociodemographic characteristics were obtained via participant self-report. Maternal sociodemographic characteristics include age (years), whether the mother has more than one child, whether the mother was in a relationship with the fathers of their children (some/all of the time) and caregiver status. Additional characteristics include maternal age at birth (years; self-report corroborated with child dates of birth from child medical records on a case-by-case basis) and maternal HIV status (obtained through self-report and corroborated with clinical notes on a case-by-case basis).

*Maternal mental health* was assessed using four validated mental health screening scales (see below). All mothers in the sample responded to all four screening measures. Cut-off scores were used to indicate experience of poor mental health across each measure. Two composite measures, any likely *common mental disorder* (scoring above the cut-off on at least one measure of mental health symptomology) and *mental health comorbidities* (scoring above the cut-off on at least two measures of mental health symptomology),^10 44^ were used to explore overall mental health status. *Depressive symptomology* was measured using the 10-item Child Depression Inventory short form.^45 46^ Scores of ≥3 (based on definitive symptoms; 0–10) were used to indicate symptomology consistent with a positive screen for depression (binary; yes/no).^47^ *Anxiety symptomology* was measured using an abbreviated version (14 items) of the Children’s Manifest Anxiety Scale—Revised (scored 0–14).^48 49^ Scores ≥10 were used to indicate symptomology consistent with a positive screen for anxiety.^48 49^ *Post-traumatic* *stress symptomology* was measured using a 12-item version of the Child post-traumatic stress disorder checklist.^50^ Four domains of post-traumatic stress disorder are represented in the items (re-experience, avoidance, hyperarousal and dysphoria).^50^ Participants were classified as experiencing symptomology consistent with a partial screen for post-traumatic stress disorder if they scored on at least one item across all four of the domains with affirmative responses (ie, ‘most of the time’/‘all of the time’; affirmative scores ranged from 0 to 12). *Suicidality/self-harm symptomology* was measured using the five-item Mini International Neuropsychiatric Interview (MINI-KID; scored 0–5).^51^ Participants were classified as reporting suicidal symptoms if they scored on any item on the MINI-KID.^51^

### Children born to adolescent mothers

Child sociodemographic characteristics including age (months) and biological sex were routinely collected from adolescent mother/caregiver reports and corroborated with child medical records on a case-by-case basis. *Child cognitive development* was assessed using the Mullen Scales of Early Learning,^42^ administered by a trained interviewer on a single occasion. Children were scored on numerous assessments relating to five developmental domains (gross motor skills, fine motor skills, visual reception, expressive language and receptive language). Raw scores were transformed to age-standardised t-scores (range 20–80). T-scores for four developmental domains were combined to create a composite score of generalised cognitive functioning (age-standardised scores ranged from 49 to 155).^42^ Based on standardised testing procedure, only children ≤39 months were eligible to complete the gross motor assessment.^32^ All other domains were completed by all children in the sample. The Mullen Scales of Early Learning have been found to have good psychometric properties and have been used extensively across sub-Saharan Africa and within South Africa.^52^

### Statistical analyses

Stata V.15^53^ was used to undertake all analyses. χ^2^ tests and t-tests (Fisher’s exact/Kruskal-Wallis tests, where appropriate) were used to explore sample characteristics according to paternal age, father involvement, maternal HIV status and maternal mental health status. Where appropriate, participants with missing data were excluded from analyses. A strength of these data was the limited number of variables with missing data.

## Results

### Sociodemographic characteristics

The median age of adolescent mothers at the birth of their first child was 17 years (IQR: 16–18 years). A quarter (25.4%; 227/894) of the adolescent mothers in the sample were living with HIV. 6.9% of adolescent mothers had given birth to more than one child, and 85.5% of mothers reported that they were the main caregivers of their child(ren). The median age of fathers of children born to adolescent mothers at the time of the child’s birth was 20 years (IQR: 18–23 years (range: 13–40 years)). Over half of adolescent mothers did not know the HIV status of the fathers of their children (53.5%; 478/894), and over half were in a relationship with the fathers of their children (52.6%; 470/894). Most fathers knew of their paternity status (98.1%; 877/894). Less than 1% of fathers were the main caregivers of their children (0.5%; 4/894). Very few (4.9% (44/894)) fathers lived with their children and the mothers of their children. 3.9% (35/894) of adolescent mothers reported that they had experienced domestic violence perpetrated by the fathers of their child(ren). A small proportion of fathers attended the first antenatal appointment with adolescent mothers (1.9%; 17/894). 84.7% (757/894) of fathers had a positive reaction to the pregnancy. The children born to adolescent mothers in the sample had a median age of 14 months (IQR: 6–27 months; see table 1).

**Table 1:** Maternal, paternal and child characteristics according to paternal age (n=894)

	N (%)/mean (SD)/median (IQR)			t/χ^2^, p-value
	Total sample (n=894)	Adolescent fathers (n=358)	Older fathers (n=536)	
Maternal characteristics				
Maternal age (years)	18 (17–19)	18 (16–19)	19 (18–19)	**95.3, 0.0001**
Maternal age at birth of child (years)	17 (16–18)	16 (15–17)	17 (16–18)	**112.8, 0.0001**
Maternal HIV status (living with HIV)	227 (25.4%)	57 (15.9%)	170 (31.7%)	**28.3, <0.001**
Mother has more than one child	62 (6.9%)	22 (6.2%)	40 (7.5%)	0.57, 0.45
Mother is the main caregiver for the child	764 (85.5%)	293 (81.8%)	471 (87.9%)	11.81, 0.07
Any common mental disorder*	110 (12.3%)	38 (10.6%)	72 (13.4%)	1.58, 0.21
Any mental health comorbidities†	24 (2.7%)	9 (2.5%)	15 (2.8%)	0.07, 0.80
Any depressive symptoms	72 (8.1%)	19 (5.3%)	53 (9.9%)	**6.08, 0.01**
Any anxiety symptoms	5 (0.6%)	0 (0.0%)	5 (0.9%)	3.36, 0.07
Any post-traumatic stress symptoms	6 (0.7%)	2 (0.6%)	4 (0.8%)	0.11, 0.74
Any suicidality symptoms	56 (6.3%)	27 (7.5%)	29 (5.4%)	1.66, 0.20
Paternal characteristics				
Paternal age (current, years)	22 (20–25)	20 (19–21)	24 (22–26)	**460.6, 0.0001**
Paternal age at birth of child (years)	20 (18–23)	18 (17–19)	22 (21–25)	**643.2, 0.0001**
Unknown father HIV status	478 (53.5%)	206 (57.4%)	272 (50.8%)	**16.62,<0.0001**
In a relationship with the child’s mother	470 (52.6%)	190 (53.1%)	280 (52.2%)	0.97, 0.62
Father knows paternity status	877 (98.1%)	349 (97.5%)	528 (98.5%)	1.20, 0.27
Father is the main caregiver for the child	4 (0.5%)	2 (0.6%)	2 (0.4%)	0.17, 0.68
Father lives with mother and child	44 (4.9%)	7 (2.0%)	37 (6.9%)	**13.8, 0.008**
Domestic violence perpetration	35 (3.9%)	13 (3.6%)	22 (4.1%)	0.13, 0.72
Father engagement				
Father looks after child	66 (7.9%)	21 (6.3%)	45 (9.0%)	1.95, 0.16
Father helps buy things for the child	153 (18.3%)	54 (16.2%)	99 (19.7%)	1.66, 0.20
Father helps with washing/food preparation for the child	56 (6.7%)	21 (6.3%)	35 (7.0%)	0.14, 0.70
Father spends time with the child	84 (10.0%)	29 (8.7%)	55 (10.9%)	1.13, 0.29
Any involvement	163 (19.5%)	57 (17.1%)	106 (21.1%)	2.06, 0.15
Involvement score (0–4)	0.43 (1.03)	0.37 (0.98)	0.47 (1.06)	1.25, 0.21
Mother and father argue about money	166 (18.6%)	50 (14.0%)	116 (21.6%)	**8.36, 0.004**
Father attended first antenatal appointment	17 (1.9%)	4 (1.1%)	13 (2.4%)	1.97, 0.16
Father had a positive reaction to pregnancy	757 (84.7%)	307 (85.8%)	450 (84.0%)	0.53, 0.46
Child characteristics				
Child age (months)	14 (6–27)	13 (5–28)	15 (7–27)	0.59, 0.44
Biological sex (female)	429 (48.0%)	176 (49.2%)	253 (47.2%)	0.33, 0.57
Child cognitive development				
Gross motor skills score	49.7 (12.7)	49.0 (12.3)	50.2 (12.9)	1.29, 0.20
Visual reception skills score	42.4 (14.3)	43.6 (14.7)	41.5 (14.0)	**−2.17, 0.03**
Fine motor skills score	44.1 (14.7)	45.2 (13.9)	43.5 (15.2)	−1.68, 0.09
Receptive language skills score	47.8 (13.5)	48.6 (13.4)	47.3 (13.6)	−1.50, 0.13
Expressive language skills score	51.8 (13.4)	52.2 (13.5)	51.6 (13.3)	−0.58, 0.56
Composite score of early learning‡	93.9 (21.4)	96.6 (21.5)	92.8 (21.2)	−1.92, 0.06

Bold denotes p-value significance level of less than 0.05 (p=<0.05)

* Common mental disorder: scoring above the cut-off on one or more screening measures for mental health.

† Mental health comorbidities: scoring above the cut-off on two or more screening measures for mental health. Missing data: any involvement/involvement score (n=837), gross motor skills score (n=798).

‡ Early learning composite score: age-standardised composite score of fine motor, visual reception, expressive language, receptive language domains (range 49–155).

### Adolescent fatherhood

40% (358/894) of fathers were adolescents (10–19 years) at the time of the birth of their children. Mothers of children born to adolescent fathers were more likely to be younger (16 vs 17 years; χ^2^=112.8, p=0.0001) and less likely to be living with HIV (15.9% vs 31.7%; χ^2^=28.3, p≤0.0001) compared with mothers of children born to older fathers. Mothers of children born to adolescent fathers were less likely to know the HIV status of the fathers compared with older fathers (57.4% vs 50.8%, χ^2^=p≤0.0001), to live with the fathers of their children (2.0% vs 6.9%, χ^2^=13.8, p=0.008) and to argue about money with the fathers of their children (14.0% vs 21.6%, X2=8.36, p≤0.004). Similar prevalence rates were identified between adolescent fathers and older fathers relating to: being in a relationship with the mothers, knowing of their children, being the main caregivers for their children, being involved with their children, reaction to pregnancy and domestic violence perpetration.

Children born to adolescent fathers were found to have higher visual reception scores compared with older fathers (43.6 vs 41.5, t=−2.17, p=0.03). Scores on all other child cognitive development domains, including the composite measure of early learning, were found to be similar regardless of father age (see table 1).

### Father involvement

19.5% (163/837) of fathers were involved with their children (at least once every 2 weeks; see table 1). Mothers of children who had involved fathers were less likely to report being the main caregivers of their children compared with fathers who were not involved with childcare (85.9% vs 90.2%, χ^2^=19.0, p=0.002). Involved fathers were more likely to be older (21 vs 20 years, t=4.30, p=0.04), to be in a relationship with the mothers of their children (74.8% vs 47.2%, χ^2^=40.8, p≤0.0001), to live with their children (14.7% vs 3.0%, χ^2^=49.3, p<0.0001) and to be the main caregivers of their children compared with noninvolved fathers (2.5% vs 0.%, p≤0.0001). Mothers of children with involved fathers were less likely to know the HIV status of their child’s father compared with noninvolved fathers (68.7% vs 48.4%, χ^2^=25.6, p≤0.0001). Involved fathers were also more likely to attend the first antenatal appointment (4.3% vs 1.5%, χ^2^=5.21, p=0.02) and to have a positive reaction to the adolescent mothers’ pregnancy (96.3% vs 81.2%, χ^2^=22.6, p≤0.0001). Children of involved fathers were found to score lower on domains of gross motor (47.6 vs 50.1, t=2.20, p=0.03) and fine motor skills (41.9 vs 44.5, t=2.07, p=0.04) compared with children of noninvolved fathers. Scores on all other child cognitive domains, including the composite measure of early learning, were found to be similar regardless of father involvement (see table 2).

**Table 2:** Maternal, paternal and child characteristics according to paternal involvement (n=837)

	N (%)/mean (SD)/median (IQR)		t/χ^2^, p value
	Any father involvement (n=163)	No father involvement (n=674)	
Maternal characteristics			
Maternal age (years)	18 (17–19)	18 (17–19)	0.05, 0.83
Maternal age at birth of child (years)	17 (16–18)	17 (16–18)	1.90, 0.17
Maternal HIV status (living with HIV)	46 (28.2%)	172 (25.5%)	0.50, 0.48
Mother has more than one child	13 (8.0%)	46 (6.8%)	0.26, 0.61
Mother is the main caregiver for the child	140 (85.9%)	608 (90.2%)	**19.0, 0.002**
Any common mental disorder*	19 (11.7%)	84 (12.5%)	0.08, 0.78
Any mental health comorbidities†	2 (1.2%)	21 (3.1%)	1.75, 0.28
Any depressive symptoms	13 (8.0%)	57 (8.5%)	0.04, 0.84
Any anxiety symptoms	1 (0.6%)	4 (0.6%)	0.0009, 1.00
Any post-traumatic stress symptoms	0 (0.0%)	5 (0.7%)	1.22, 0.59
Any suicidality symptoms	8 (4.9%)	42 (6.2%)	0.41, 0.52
Paternal characteristics			
Paternal age (current, years)	23 (20–25)	22 (20–25)	1.32, 0.25
Paternal age at birth of child (years)	21 (19–23)	20 (18–23)	**4.30, 0.04**
Adolescent father (10–19 years)	57 (35.0%)	277 (41.1%)	2.06, 0.15
Unknown father HIV status	112 (68.7%)	326 (48.4%)	**25.6, <0.0001**
In a relationship with the child’s mother	122 (74.8%)	318 (47.2%)	**40.8, <0.0001**
Father is the main caregiver for the child	4 (2.5%)	0 (0.0%)	**n/a**
Father lives with mother and child	24 (14.7%)	20 (3.0%)	**49.3, <0.0001**
Domestic violence perpetration	7 (4.3%)	25 (3.7%)	0.12, 0.73
Father engagement			
Mother and father argue about money	26 (16.0%)	127 (18.8%)	0.73, 0.39
Father attended first antenatal appointment	7 (4.3%)	10 (1.5%)	**5.21, 0.02**
Father had a positive reaction to pregnancy	157 (96.3%)	547 (81.2%)	**22.6, <0.0001**
Child characteristics			
Child age (months)	13 (5–26)	14 (6–28)	0.69, 0.41
Biological sex (female)	80 (49.1%)	318 (47.2%)	0.19, 0.66
Child development scores			
Gross motor skills score	47.6 (11.6)	50.1 (12.9)	**2.20, 0.03**
Visual reception skills score	41.8 (14.8)	42.6 (14.2)	0.63, 0.53
Fine motor skills score	41.9 (14.3)	44.5 (14.8)	**2.07, 0.04**
Receptive language skills score	47.2 (13.9)	48.1 (13.5)	0.70, 0.48
Expressive language skills score	52 (13.4)	51.6 (13.5)	0.41, 0.68
Composite score of early learning‡	94.4 (21.1)	92.2 (22.8)	1.18, 0.24

Note: Father involvement data available for n=837. If fathers help look after the child, buy things for the child, help with washing and/or food preparation or spend time with the child at least once every 2 weeks, they were classified as ‘involved’ for the purpose of analyses. Missing data: Gross motor skills score (n=748).

Bold denotes p-value significance level of less than 0.05 (p=<0.05)

* Common mental disorder: scoring above the cut-off on one or more screening measures for mental health.

† Mental health comorbidities: scoring above the cut-off on two or more screening measures for mental health.

‡ Early learning composite score: age-standardised composite score of fine motor, visual reception, expressive language, receptive language domains (range 49–155).

### Fatherhood, maternal HIV and maternal mental health

#### Father characteristics according to adolescent maternal HIV status

Fathers of children born to adolescent mothers living with HIV were more likely to be older and less likely to be adolescent fathers compared with fathers of children born to adolescent mothers not living with HIV (25.1% vs 45.1%, χ^2^=28.3, p≤0.0001). Adolescent mothers living with HIV were more likely to know the HIV status of the fathers of their children compared with adolescent mothers not living with HIV (unknown father HIV status: 15.0% vs 66.6%, χ^2^=260.9, p≤0.0001) and more likely to be living with the fathers of their children (8.8% vs 3.6%, χ^2^=23.7, p≤0.0001). Fathers of children born to adolescent mothers living with HIV were more likely to have a positive reaction to the pregnancy compared with fathers of children born to adolescent mothers not living with HIV (89.0% vs 83.2%, χ^2^=4.36, p=0.04; see table 3).

**Table 3:** Paternal characteristics according to maternal HIV status (n=894)

	N (%)/mean (SD)/median (IQR)		t/χ^2^, p value
	Adolescent mothers living with HIV (n=227)	Adolescent mothers not living with HIV (n=667)	
Paternal characteristics			
Paternal age (current, years)	25 (22–28)	21 (20–24)	**92.3, 0.0001**
Paternal age at birth of child (years)	22 (19–25) Range: 14–38	20 (18–22) Range: 13–40	**58.1, 0.0001**
Adolescent father (10–19 years)	57 (25.1%)	301 (45.1%)	**28.3, <0.0001**
Unknown father HIV status	34 (15.0%)	444 (66.6%)	**260.9, <0.0001**
In a relationship with the child’s mother	118 (52.0%)	352 (52.8%)	3.76, 0.15
Father knows paternity status	224 (98.7%)	653 (97.9%)	0.55, 0.46
Father is the main caregiver for the child	1 (0.4%)	3 (0.5%)	0.0003, 0.99
Father lives with mother and child	20 (8.8%)	24 (3.6%)	**23.7, <0.0001**
Domestic violence perpetration	11 (4.9%)	24 (3.6%)	0.70, 0.40
Father engagement			
Father looks after child	18 (8.3%)	48 (7.8%)	0.06, 0.81
Father helps buy things for the child	43 (19.7%)	110 (17.8%)	0.41, 0.52
Father helps with washing/food preparation for the child	15 (6.9%)	41 (6.6%)	0.02, 0.90
Father spends time with the child	25 (11.5%)	59 (9.5%)	0.67, 0.41
Any involvement	46 (21.1%)	117 (18.9%)	0.50, 0.48
Involvement score (scored 0–4)	0.46 (1.06)	0.42 (1.02)	0.23, 0.63
Mother and father argue about money	50 (22.0%)	116 (17.4%)	2.41, 0.12
Father attended first antenatal appointment	5 (2.2%)	12 (1.8%)	0.15, 0.70
Father had a positive reaction to pregnancy	202 (89.0%)	555 (83.2%)	**4.36, 0.04**

Note: Missing data: any involvement/involvement score (n=837).

Bold denotes p-value significance level of less than 0.05 (p=<0.05)

#### Father characteristics according to adolescent maternal mental health status

12.3% (110/894) of adolescent mothers reported symptoms of a likely common mental disorder (see table 1). Mothers of children born to adolescent fathers were less likely to report depressive symptoms compared with older fathers (5.3% vs 9.9%; χ^2^=6.08, p=0.01). The prevalence rates of all other mental health symptoms were found to be similar among mothers of adolescent and older fathers (see table 1). The prevalence of mental health symptomology was similar regardless of fatherhood involvement status (see table 2).

Compared with mothers not classified as experiencing likely common mental disorder, mothers classified as experiencing likely common mental disorder were more likely to know the HIV status of the fathers of their child(ren) (unknown HIV status: 40.0% vs 55.4%, χ^2^=13.3, p=0.001), to be in a relationship with the father of their firstborn child (41.8% vs 54.1%, χ^2^=13.3, p=0.03), to report domestic violence perpetrated by the fathers of their children (8.2% vs 3.3%, t=6.07, p=0.01) and to argue about money with the fathers of their children (30.0% vs 17.0%, χ^2^=10.8, p=0.0001; see table 4).

**Table 4:** Paternal characteristics according to maternal mental health status (n=894)

	N (%)/mean (SD)/median (IQR)		t/χ^2^, p value
	Any common mental disorder (n=110)	No common mental disorder (n=784)	
Paternal characteristics			
Paternal age (current, years)	23 (21–26)	22 (20–25)	**5.02, 0.03**
Paternal age at birth of child (years)	21 (19–23)	20 (18–23)	1.25, 0.27
Adolescent father (10–19 years)	38 (34.6%)	320 (40.8%)	1.58, 0.21
Unknown father HIV status	44 (40%)	434 (55.4%)	**13.3, 0.001**
In a relationship with the child’s mother	46 (41.8%)	424 (54.1%)	**7.32, 0.03**
Father knows paternity status	110 (100%)	767 (2.17%)	2.43, 0.12
Father is the main caregiver for the child	0 (0.0%)	4 (0.5%)	0.56, 0.45
Father lives with mother and child	5 (4.6%)	39 (5.0%)	3.10, 0.54
Domestic violence perpetration	9 (8.2%)	26 (3.3%)	**6.07, 0.01**
Father engagement			
Father looks after child	8 (7.8%)	58 (7.9%)	0.002, 0.96
Father helps buy things for the child	16 (15.5%)	137 (18.7%)	0.60, 0.44
Father helps with washing/food preparation for the child	5 (4.9%)	51 (7.0%)	0.63, 0.43
Father spends time with the child	11 (10.7%)	73 (10.0%)	0.05, 0.82
Any involvement	19 (18.5%)	144 (19.6%)	0.08, 0.78
Involvement score (scored 0–4)	0.39 (0.96)	0.43 (1.04)	0.43, 0.67
Mother and father argue about money	33 (30.0%)	133 (17.0%)	**10.84, 0.001**
Father attended first antenatal appointment	4 (3.6%)	13 (1.7%)	2.02, 0.16
Father had a positive reaction to pregnancy	90 (81.8%)	667 (86.1%)	0.79, 0.38

Note: Missing data: Any involvement/involvement score (n=837).

Bold denotes p-value significance level of less than 0.05 (p=<0.05)

## Discussion

This study provides the first detailed exploration of the characteristics of fathers of children born to adolescent mothers in the context of HIV in South Africa. Analyses provide a descriptive summary of the characteristics of fathers of children born to adolescent mothers, based on adolescent mother reports, as a foundation for future targeted research studies on the topics of interest. Overall, father involvement was low (19.5% of fathers were involved with the care of their children at least once every 2 weeks, and 0.5% of fathers were the main caregivers of their children). The prevalence of adolescent versus adult fatherhood was high (40%). Adolescent fatherhood was linked to younger maternal age, lower prevalence of maternal HIV, fathers being less likely to live with their children and lower prevalence of arguments between adolescent mothers and fathers about finances. Involved fathers were more likely to be older, in a relationship with adolescent mothers and to live with their children. The fathers of children born to adolescent mothers living with HIV were more likely to be older, to live with their children and to have a positive reaction to pregnancy. Poor maternal mental health was linked to not being in a relationship with the father, heightened domestic violence perpetrated by the fathers of their children and financial arguments.

A greater understanding of barriers to father involvement is required. Efforts to bolster father engagement, such as the inclusion of fathers within maternal and child service provision, may have benefits for fathers, adolescent mothers and their children. Antenatal care needs a refocus to welcome and include fathers and even to evolve to offer healthcare and benefits directly to the father when attending such venues.^54^ Adolescent fathers may have specific needs requiring tailored intervention to support and promote the well-being of adolescent parent families. Mental health support and existing HIV services may be an important access point to these families with benefits for parents and their child(ren). There remains a critical need for the inclusion of fathers within policy, programming and research agendas.

These findings provide an initial exploration of the prevalence rates of adolescent fatherhood within South Africa and a timely contribution to the literature focusing on adolescent fathers.^30^ In support of previous studies emerging from South Africa, father involvement within the sample was low.^20 55 56^ Uniquely, this study explores father involvement in several ways (involvement with their children, whether they are the main caregivers and engagement with antenatal appointments). Involved fathers were more likely to attend the first antenatal appointment, highlighting the importance of encouraging father involvement from the early stages of pregnancy. A small number of fathers lived with their children (<5%) which was lower than the national prevalence in South Africa (among both adolescent and adult fathers; 35.6%).^57^ Adolescent fathers had lower levels of involvement compared with adult fathers. Further research is required to explore barriers to father engagement (inclusive of adolescent father engagement) within the South African context. Possible barriers may include aspects of the cultural context, gender stereotypes, poverty/financial constraints and a lack of employment opportunities resulting in fathers migrating away from their children and the exclusion of fathers from services and programming inclusive of health and education.^16^ Bolstering positive father involvement with their children and throughout pregnancy may be of benefit to adolescent mothers, their children and fathers themselves.^2022 23 58^,^60^

In contrast to previous literature, while some differences in individual cognitive development subscale scores were identified according to father age and father involvement, no differences in overall composite early learning scores were identified. Previous research has identified older father age (nonadolescent fathers) and father involvement as being beneficial for child development outcomes. These data provide an initial exploration of these factors. Seemingly, the relationship between fatherhood and child cognitive development scores within the context of adolescent pregnancy and HIV may be more complex than these initial investigations. Living with HIV or being exposed to HIV has been previously found to have impacts on child cognitive development. These analyses did not exclude the small number of children (n=10) known to be living with HIV in the sample. The cognitive development scores of these children may have impacted on findings; however, any differences are likely to be minimal given the small sample of children known to be living with HIV. However, difficulties with cognitive development may have warranted increased involvement of fathers. Further research exploring the longitudinal impacts of fatherhood characteristics on the cognitive development of children born to adolescent mothers affected by HIV, inclusive of mediating and moderating factors (ie, child HIV exposure status, socioeconomic status), is required to expand our understanding and meaningfully contribute to beneficial policy and programming for this group.

Findings additionally provide rare insight into fatherhood characteristics within the context of adolescent pregnancy and HIV and support previous literature identifying a higher prevalence of HIV among adolescent girls with older sexual partners. Adolescent mothers remain disproportionately at risk of HIV infection compared with their non-mother counterparts^8^—with HIV incidence risk up to five times greater among this group.^61^ As such, adolescent mothers with children born to older fathers are a particularly vulnerable group who may require intervention to reduce HIV risk. Those adolescent mothers who are living with HIV require appropriate testing, care and treatment inclusive of prevention of vertical transmission.^11^

### Strengths and limitations

While there is a growing body of evidence exploring maternal mental health within the context of adolescent pregnancy and HIV in sub-Saharan Africa, the mental health experience of adolescent mothers remains largely unknown. These are the first analyses to explore such concepts within the context of fatherhood. Within the sample, the prevalence of poor maternal mental health symptoms was similar regardless of father age or involvement. Findings support previous research highlighting relationship status, violence exposure and domestic disputes as being possibly linked to mental health and expand such literature to include adolescent mothers affected by HIV in South Africa. Mental health support and interventions may be an important access point for adolescent parent families, with benefits for adolescent mothers, fathers and their children.

Study limitations should be considered within the interpretation of results. First, the data presented are cross-sectional, and therefore it remained difficult to establish the direction of causality. Second, these data are exploratory and thus only provide initial insight into the characteristics of children born to adolescent mothers. Analyses explore only a small number of characteristics, and factors that may mediate or moderate any differences between groups identified within analyses have not been explored. Additionally, interactions between maternal characteristics (eg, maternal mental health and HIV status) have not been explored. As such, these analyses present a partial view of fatherhood within the context of adolescent motherhood and provide a foundation for future research within this topic area. Third, data are drawn from adolescent mother reports. Thus, the findings are confined to maternal views and subsequent research would benefit from data provided by fathers themselves. Data are drawn from the Eastern Cape Province of South Africa, potentially restricting the generalisability of results beyond the region. Despite these limitations, these initial findings are drawn from a robust sample of adolescent mothers within South Africa and offer one of the first explorations of fatherhood within the context of adolescent motherhood and HIV.

## Conclusions

Adolescent mothers and their children affected by HIV remain substantially underserved with regard to policy and programming and are often neglected within research agendas. These analyses aimed to address this critical evidence gap, promote the notion of fathers within this context and describe the characteristics of fathers of children born to adolescent mothers affected by HIV. Father involvement with their children was low in the sample, and the prevalence of adolescent fatherhood was high. Characteristics of fathers had possible impacts on both adolescent mothers and their children. Despite their exploratory nature, these analyses have implications for the design of interventions, policy and programming for adolescent parent families in South Africa and provide a foundation for future research. Maternal HIV, maternal mental health and child development should remain key considerations in future investigations. Efforts to bolster father engagement, such as the inclusion of fathers within maternal and child service provision, may have benefits for fathers, adolescent mothers and their children. However, services and programming may require specific adaptations to meet the needs of adolescent fathers.

## Data Availability

Data are available upon reasonable request.
