# Supplementary material for: Men matter: a cross-sectional exploration of the forgotten fathers of children born to adolescent mothers in South Africa
Source: BMJ Open. 2025 Jul 3;15(7):e092723. doi: 10.1136/bmjopen-2024-092723 (PMC12226945; doi:10.1136/bmjopen-2024-092723)
Supplement: Supplementary file 1 [file bmjopen-2024-092723.reviewer_comments.pdf]

PEER REVIEW HISTORY

BMJ Open publishes all reviews undertaken for accepted manuscripts. Reviewers are asked to complete a checklist review form and are provided with free text boxes to elaborate on their assessment. These free text comments are reproduced below.

ARTICLE DETAILS

Title (Provisional)

Men matter: A cross-sectional exploration of the forgotten fathers of children born to adolescent mothers in South Africa

Authors

Steventon Roberts, Kathryn; Smith, Colette; Cluver, Lucie Dale; Toska, Elona; Kelly, Jane; Thomas, Angelique; Tolmay, Janke; Marlow, Marguerite; Sherr, Lorraine

---

VERSION 1 - REVIEW

**Reviewer**	**1**
**Name**	**Bulterys, Michelle**
**Affiliation**	**University of Washington, Department of Epidemiology**
**Date**	**12-Nov-2024**
**COI**	**None**

This is an excellent manuscript - thank you for highlight such an important, yet neglected, topic of research. Please find some minor comments for your consideration.

Abstract:

- Worth adding in the abstract that the father characteristics were self-reported by the adolescent mothers.

- How was father involvement defined?

- Were HIV and depressive symptoms correlated with each other? Did you assess interaction between the two, with regard to father characteristics?

- Excellent conclusion

Introduction:

- "It is well established that active father involvement has broad positive impacts for both child13-19 (i.e. development, feeding) and maternal outcomes (i.e. mental health).20-23" I suggest adding/citing literature here of the beneficial role of father involvement on improved maternal PMTCT outcomes as well (ART adherence, viral suppression, and infant HIV-free survival).

- Is there literature on whether adolescent girls and young women are more likely to acquire HIV from older men in this setting?

- Could be interesting to summarize some findings by Sonke Gender Justice regarding father absence in South Africa "So we are ATM fathers".

- "Father involvement may also interrupt intergenerational cycles of adolescent pregnancy and parenting…" – Is this in regards to pregnant girls' fathers, or male partners (fathers of their baby)? I imagine both definitions of fathers would be important, but perhaps the distinction can be made clearer here.

Methods:

- How was "father" defined? To confirm, mothers were asked specifically to provide details about the father of their current baby, not just any male partner? What if another male partner has taken over the role as a "father"?

- If relevant, could you please provide additional details on the piloting process, and any changes that were made because of that process?

- How different were the categories "father looks after child" and "father spends time with child"? Did responses to these overlap quite closely?

- How was domestic violence perpetuation collected? Over what time period?

- How often did children undergo the Mullen assessments?

Results:

- Almost all fathers were aware of the pregnancy, however <5% lived together. Is this similar/expected in other cohorts in South Africa or is this especially low?

- "Children of involved fathers were found to score lower on domains of gross motor". Why could this be?

Discussion:

- "Maternal mental health was linked to being in a relationship with the father, heightened domestic violence perpetrated by the father of their child, and financial arguments." Could you clarify – should this be phrased as poor maternal mental health?

- "Living with HIV or being exposed to HIV has been previously found to have impacts on chid cognitive development". Please correct the typo in "child".

- Could you compare your prevalence of paternal involvement to literature around paternal absence in South Africa, or Southern African regions (not adolescent father-specific)? Why do you think involvement is lower among adolescent fathers compared to adult fathers? Financial/living constraints? More discussion on this phenomenon would be helpful and interesting.

**Reviewer**	**2**
**Name**	**olowookere, samuel**
**Affiliation**	**Obafemi Awolowo University**
**Date**	**22-Apr-2025**
**COI**	**None**

Good manuscript. The abstract conclusion appear too long and can be reduced

VERSION 1 - AUTHOR RESPONSE

Reviewer: 1
Dr. Michelle Bulterys, University of Washington
Comments to the Author:
This is an excellent manuscript - thank you for highlight such an important, yet neglected, topic of research. Please find some minor comments for your consideration.

Thank you for your comment and for taking the time to review the manuscript.

Abstract:
- Worth adding in the abstract that the father characteristics were self-reported by the adolescent mothers.

Further detail has been added for clarity. "Summary statistics are presented based on maternal self-report of father characteristics"

- How was father involvement defined?

Detail has been added and the definition of father involvement is outlined in detail in the methodology section.

"Chi-square tests, t-test (Fisher's exact/Kruskal Wallis tests, where appropriate) were additionally used to explore sample characteristics (including father characteristics, maternal experience, and child characteristics) according to paternal age and father involvement in childcare (defined by responses to 4 maternal self-report questions)."

- Were HIV and depressive symptoms correlated with each other? Did you assess interaction between the two, with regard to father characteristics?

Thank you – this is an interesting query! Within the broader study, adolescent mothers living with HIV were more likely to report depressive symptoms (11.4% vs. 6.9%). This interaction was not assessed within this manuscript in relation to father characteristics

given the focus on broader father experience, the overlap in other analyses being undertaken as part of the broader study and the space needed to unpack the elements of mental health fully. However, a note has been added to the limitations section of the manuscript in relation to this comment. Thank you again – we hope to explore this further in some upcoming analyses.

"Analyses explore only a small number of characteristics and, factors which may mediate or moderate any differences between groups identified within analyses have not been explored. Additionally, interactions between maternal characteristics (e.g., maternal mental health and HIV status) have not been explored. As such, these analyses present a partial view of fatherhood within the context of adolescent motherhood and provide a foundation for future research within this topic area"

- Excellent conclusion

Thank you.

Introduction:
- "It is well established that active father involvement has broad positive impacts for both child13-19 (i.e. development, feeding) and maternal outcomes (i.e. mental health).20-23" I suggest adding/citing literature here of the beneficial role of father involvement on improved maternal PMTCT outcomes as well (ART adherence, viral suppression, and infant HIV-free survival).

Further detail and references have now been added. Thank you for this comment.

"It is well established that active father involvement has broad positive impacts for both child[13-19] (i.e. development, feeding), maternal outcomes (i.e. mental health) and prevention of mother to child transmission outcomes (i.e., antiretroviral adherence, viral suppression and HIV-free infant survival)."

- Is there literature on whether adolescent girls and young women are more likely to acquire HIV from older men in this setting?

References and further detail has now been added.

"Much of the existing literature on fatherhood focuses on adult fathers, with particular attention to age disparate relationships when it comes to teenage mothers. This age disparity raises issues of concerns of abuse, transactional sex, increased HIV acquisition, and child marriage."

- Could be interesting to summarize some findings by Sonke Gender Justice regarding father absence in South Africa "So we are ATM fathers".

Further detail from this report has now been added to the introduction section of the manuscript.

"Both wanting to demonstrate masculinity and engaging in sexual risk-taking behaviours have been identified as factors contributing to adolescent fatherhood in South Africa.[29] Challenges for adolescent fatherhood include financial dependency and caring responsibilities for children.[29] Previous research in South Africa has highlighted the prevalent perception among fathers, and often mothers, that a father's primary role is to provide maternal provision. If fathers cannot provide financially (especially due to high unemployment rates), this may lead to a sense of emasculation and inadequacy, prompting fathers to limit their involvement (of have their involvement limited) with their children"

- "Father involvement may also interrupt intergenerational cycles of adolescent pregnancy and parenting…" – Is this in regards to pregnant girls' fathers, or male partners (fathers of their baby)? I imagine both definitions of fathers would be important, but perhaps the distinction can be made clearer here.

Some additional detail has been added for clarity.

"Father involvement (referring to the fathers of children born to adolescent mothers) may be a critical form of support for adolescent mothers and their children. Father presence may also interrupt intergenerational cycles of adolescent pregnancy and parenting, as father absenteeism within South Africa has previously been linked to early sexual debut and adolescent pregnancy.[33]"

With reference to this sentence…

"Father presence may also interrupt intergenerational cycles of adolescent pregnancy and parenting, as father absenteeism within South Africa has previously been linked to early sexual debut and adolescent pregnancy"

We feel that both definitions of father would be valid as the fathers of adolescents may reduce adolescent pregnancy and within future generations – if a child has a present father, early sexual debut and adolescent pregnancy rates in future generation may be reduced.

Methods:

- How was "father" defined? To confirm, mothers were asked specifically to provide details about the father of their current baby, not just any male partner? What if another male partner has taken over the role as a "father"?

Clarity around the definition of father has now been added to the methodology section of the manuscript.

“ This study included data for 894 fathers based on adolescent mother report. Data were drawn from a cross-sectional study of a cohort of young mothers (10-24 years; n=1046) residing in the Eastern Cape Province, South Africa. Participants were excluded from analyses if; young mothers who were not classified as adolescent mothers (who had given birth aged 20 years and above; n=44), children were above 68 months of age (in keeping with the validated age range of the Mullen Scales of Early Learning;[38] n=23), if the fathers were unknown to the mother (hence no fatherhood reported data), or if the fathers age was unknown (n=80). Mothers were asked to provide details about the biological father of their child. This did not include male partners who were not the biological father of their child. Second and third born children were excluded from analyses relating to child cognitive development (n=5).”

- If relevant, could you please provide additional details on the piloting process, and any changes that were made because of that process?

No major changes arose as a result of the piloting process and therefore the authors felt that this was not a relevant change.

- How different were the categories “father looks after child” and “father spends time with child”? Did responses to these overlap quite closely?

The overall involvement score for the sample was 0.43 (scored 0-4) suggesting limited overlap between the involvement categories – suggesting limited overlap between categories. A note has now been added to the methodology section.

“These four questions were combined into a composite measure indicating father involvement (scored 0-4) with higher scores representative of involvement in more activities. Overall involvement score for the sample was 0.43 (SD:1.03; scored 0-4) suggesting limited overlap between involvement categories.”

- How was domestic violence perpetuation collected? Over what time period?

Additional detail has now been added to the methodology section.

“…domestic violence perpetration towards the mother (adolescent mothers were asked if they had experienced domestic violence perpetrated by the father of their child during their pregnancy)…”

- How often did children undergo the Mullen assessments?

Mullen assessments were undertaken once. Detail has now been added for clarity

"**Child cognitive development** was assessed utilising the Mullen Scales of Early Learning,[38] administered by a trained interviewer on a single occasion. "

Results:
- Almost all fathers were aware of the pregnancy, however <5% lived together. Is this similar/expected in other cohorts in South Africa or is this especially low?

Thank you for this comment. This is especially low. Further detail has now been added to the discussion section of the manuscript, along with relevant references.

"These findings provide an initial exploration for prevalence rates of adolescent fatherhood within South Africa and provide a timely contribution to the literature focusing on adolescent fathers.[27] In support of previous studies emerging from South Africa, father involvement within the sample was low.[20,50,51] Uniquely, this study explores father involvement in several ways (involvement with their child, whether they are the main caregiver and, engagement with antenatal appointments). Involved fathers were more likely to attend the first antenatal appointment, highlighting the importance of encouraging father involvement from the early stages of pregnancy. A small number of fathers lived with their child (<5%) – lower than the national prevalence in South Africa (35.6%). "

- "Children of involved fathers were found to score lower on domains of gross motor". Why could this be?

This result has now been elaborated on further in the discussion section of the manuscript.

"In contrast to previous literature, while some differences on individual cognitive development subscale scores were identified according to father age and father involvement, no differences in overall composite early learning scores were identified. Previous research has identified older father age (non-adolescent fathers) and father involvement as being beneficial for child development outcomes. These data provide an initial exploration of these factors. Seemingly, the relationship between fatherhood and child cognitive development scores within the context of adolescent pregnancy and HIV may be more complex than these initial investigations. Living with HIV or being exposed to HIV has been previously found to have impacts on child cognitive development. These analyses did not exclude the small number of children (n=10) known to be living with HIV in the sample. The cognitive development scores of these children may have impacted on findings however, any differences are likely to be minimal given the small sample of children known to be living with HIV. However, difficulties with cognitive development may have warranted increase involvement of fathers."

Discussion:
- "Maternal mental health was linked to being in a relationship with the father, heightened domestic violence perpetrated by the father of their child, and financial arguments." Could you clarify – should this be phrased as poor maternal mental health?

This sentence has now been clarified.

"Poor maternal mental health was linked to not being in a relationship with the father, heightened domestic violence perpetrated by the father of their child, and financial arguments."

- "Living with HIV or being exposed to HIV has been previously found to have impacts on chid cognitive development". Please correct the typo in "child".

Done. Thank you.

- Could you compare your prevalence of paternal involvement to literature around paternal absence in South Africa, or Southern African regions (not adolescent father-specific)? Why do you think involvement is lower among adolescent fathers compared to adult fathers? Financial/living constraints? More discussion on this phenomenon would be helpful and interesting.

Further detail has now been added to the discussion section of the manuscript.

"Involved fathers were more likely to attend the first antenatal appointment, highlighting the importance of encouraging father involvement from the early stages of pregnancy. A small number of fathers lived with their child (<5%) – lower than the national prevalence in South Africa (among both adolescent and adult fathers; 35.6%). Adolescent fathers had lower levels of involvement compared to adult fathers. Further research is required to explore barriers to father engagement (inclusive of adolescent father engagement) within the South African context. Possible barriers may include aspects of the cultural context, gender stereotypes, poverty/financial constraints and a lack of employment opportunities resulting in fathers migrating away from their child and, the exclusion of fathers from services and programming inclusive of health and education.[16] Bolstering positive father involvement with their children and throughout pregnancy may be of benefit to adolescent mothers, their children, and fathers themselves.[20,22,23,52-54]"

Reviewer: 2
Dr. samuel olowookere, Obafemi Awolowo University
Comments to the Author:
Good manuscript. The abstract conclusion appear too long and can be reduced.

Thank you for your comments and for taking the time to review. The abstract conclusion has now been reduced.
